# A Primer Lesson in “Metaphysics”

**Published:** 1885-10

**Authors:** 


### DOMESTIC CORRESPONDENCE.

A PRIMER LESSON IN "METAPHYSICS."

TO THE EDITORS OF THE *Chicago Medical Journal and Examiner.*

*Gentlemen:*

When the first sporadic cases of "metaphysics" appeared in this city, directly after its importation from Boston, the following conversation took place between our esteemed medical friend, Dr. T., and one of the most noted and successful practitioners of the art in the Northwest. Dr. T. took an early opportunity of calling on the metaphysician, with whom he had a slight acquaintance. After apologizing for the intrusion, he said:

"Doctor, have you any knowledge of medicine?"

*Metaphysician.* "No, sir; that is not necessary to the practice of our mode of healing."

*Dr. T.* "Do you know anything of anatomy and physiology?"

*Metaphysician.* "No, sir."

*Dr. T.* "What is disease?"

*Metaphysician.* "There is no disease. The body and all its functions are mere ideas; therefore so-called 'disease' is only a suffering belief. We go farther and assert that matter is a function of mind. For example: The mantel (pointing to an ordinary marble mantel) you now look at has no real existence. You fancy that it has, and add certain properties, as hardness, color, etc., as unsubstantial and unreal as the thing itself."

*Dr. T.* "How do you account for organic disease, for instance, a broken bone?"

*Metaphysician.* "The bone is not broken; the patient merely has the idea that he has sustained a fracture. After a certain course of treatment, as you term it, he recovers from this false idea and is again restored. I endeavor to at once correct the false idea."

*Dr. T.* "Since, Doctor, you have no body, and the mantel has no existence, will you kindly strike your head violently against the mantel? Of course, you will sustain no injury, as the mantel, your head and the act itself are merely ideas, and you can immediately apply the necessary correction."

*Metaphysician.* "I do not care to experiment."

*Dr. T.* "Have drugs any action on the system?"

*Metaphysician.* "Certainly not."

*Dr. T.* "Either good or bad?"

*Metaphysician.* "No."

*Dr. T.* "I have, Doctor, a substance in this vial generally considered poisonous. You can have no objection to convincing me of the truth of your assertions by swallowing a little?"

*Metaphysician.* "As I said before, I do not care to experiment."

*Dr. T.* "Good morning, Doctor."

*Metaphysician.* "Good morning."

"Metaphysics" has had an unusual rise and extension since its first advent a little more than a year ago. It has numbered many thousands of our wealthy and intelligent citizens among its advocates. One family of "practitioners" have made thousands of dollars in the past year. A prominent physician on the West Side stated that they made great inroads into his practice. The desertions were not singly or by families, but literally in squads. His patients have mostly all returned disappointed, if not wiser. A patient affected with spasm of the eyelids, due to marked unilateral myopia, was treated daily for *five months.* Another nearly lost her life from hæmorrhage due to a uterine fibroid. A man presented himself at the Woman's Medical College for a diagnosis. He said he did not want treatment, but merely came there to inform the faculty how bad his case was, that they might be convinced of the truth and success of "metaphysics." The diagnosis was phthisis in the third stage. He is dead now.

We should like to know if there is any way for the State Board of Health to reach this kind of quackery. Perhaps the best way is to let it run its course. It will soon take its place with blue glass, magnetism, spiritual healing, etc., as a curiosity of medicine. There is not and perhaps ought not to be any law to prevent a man from making a fool of himself, if he wants to. Yours truly,

MEDICUS.

